# Cell Patterning on Photolithographically Defined Parylene-C: SiO_2_ Substrates

**DOI:** 10.3791/50929

**Published:** 2014-03-07

**Authors:** Mark A. Hughes, Paul M. Brennan, Andrew S. Bunting, Mike J. Shipston, Alan F. Murray

**Affiliations:** ^1^Centre for Integrative Physiology, School of Biomedical Sciences, The University of Edinburgh; ^2^Edinburgh Cancer Research Centre, Institute of Genetics and Molecular Medicine, Western General Hospital; ^3^School of Engineering, Institute for Integrated Micro and Nano Systems, The University of Edinburgh

**Keywords:** Bioengineering, Issue 85, Receptors, Cell Surface, Polymers, Cell Adhesion, Biomedical and Dental Materials, parylene-C, silicon dioxide, photolithography, cell adhesion, Cell Patterning

## Abstract

Cell patterning platforms support broad research goals, such as construction of predefined *in vitro* neuronal networks and the exploration of certain central aspects of cellular physiology. To easily combine cell patterning with Multi-Electrode Arrays (MEAs) and silicon-based ‘lab on a chip’ technologies, a microfabrication-compatible protocol is required. We describe a method that utilizes deposition of the polymer parylene-C on SiO_2 _wafers. Photolithography enables accurate and reliable patterning of parylene-C at micron-level resolution. Subsequent activation by immersion in fetal bovine serum (or another specific activation solution) results in a substrate in which cultured cells adhere to, or are repulsed by, parylene or SiO_2_ regions respectively. This technique has allowed patterning of a broad range of cell types (including primary murine hippocampal cells, HEK 293 cell line, human neuron-like teratocarcinoma cell line, primary murine cerebellar granule cells, and primary human glioma-derived stem-like cells). Interestingly, however, the platform is not universal; reflecting the importance of cell-specific adhesion molecules. This cell patterning process is cost effective, reliable, and importantly can be incorporated into standard microfabrication (chip manufacturing) protocols, paving the way for integration of microelectronic technology.

**Figure:**
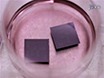


## Introduction

Understanding mechanisms that dictate cell adhesion and patterning on synthetic materials is important for applications such as tissue engineering, drug discovery, and the fabrication of biosensors^1-3^. Many techniques are available and evolving, each taking advantage of the myriad biological, chemical, and physical factors that influence cell adhesion.

Here, we describe a cell-patterning technique that utilizes processes initially developed for microelectronic fabrication purposes. As such, the platform is well-placed to enable downstream integration of microelectronic technologies, such as MEAs, into the patterning platform.

The interface between a cell membrane and an adjacent material is bi-directional and complex. *In vivo*, extracellular matrix proteins provide structure and strength and impact upon cell behavior via interactions with cell adhesion receptors. Similarly, cells *in vitro* interact with synthetic substrates via absorbed layers of proteins^4^ whilst physico-chemical influences also modulate adhesion. For example, a polymer surface can be rendered more “wettable” (hydrophilic) by ions or ultraviolet light irradiation, or etching by treatment with acid or hydroxide^5^. Established methods for cell patterning take advantage of these and other cell adhesion mediators. Examples include inkjet printing^6^, microcontact stamping^7^, physical immobilization^8^, microfluidics^9^, real-time manipulation^10^, and selective molecular assembly patterning (SMAP)^11^. Each has specific benefits and limitations. A key driver in our work, however, is to integrate cell patterning with microelectromechanical systems (MEMS).

MEMS refer to extremely small mechanical devices driven by electricity. This overlaps with the nanoscale equivalent, nanoelectromechanical systems. This concept became practical only when semiconductor strategies enabled fabrication to take place at the microscale. Microfabrication techniques developed originally for semiconductor electronics have inadvertently been found useful for other uses such as cellular electrophysiology, for example. A key downstream aim is to combine such microelectronic technologies with a high fidelity cell patterning process (forming a bioMEMS device). Several existing and otherwise reliable and practical cell-patterning techniques are incompatible with this idea. For example, accurate alignment of any embedded microelectronics or biosensors is fundamental to their efficacy but is extremely difficult to achieve using a technique such as microcontact stamping.

To circumvent this problem, we are working on a SiO_2_-based patterning platform that uses photolithographically printed parylene-C. Photolithography involves transfer of geometric features from a mask to a substrate via UV illumination. A mask is designed using an appropriate computer-aided design program. Upon a glass plate, a thin layer of nontransparent chromium represents the desired geometric pattern (a feature resolution of 1-2 mm is possible). The substrate to be patterned is coated with a thin layer of photoresist (a UV-sensitive polymer). The coated polymer is then aligned and brought into close contact with the mask. A UV source is applied such that unprotected areas are irradiated and therefore become soluble and removable in the next development step, leaving a parylene-C representation of the mask pattern behind. This process originated during development of semiconductor devices. As such, silicon wafers are frequently used as a substrate. Photolithographic deposition of parylene-C on SiO_2_ is hence a straightforward and reliable process that routinely takes place in microelectronic cleanroom facilities.

Whilst parylene has several desirable bioengineering characteristics (chemically inert, non bio-degradable), a factor restricting its *direct* use in cell patterning is its innately poor cell adhesiveness, attributed in part to its extreme hydrophobicity. Nevertheless, parylene-C has previously been used indirectly for cell patterning, for example as a peel-away cellular template^12,13^. This approach is limited by poor resolution and requires multiple steps. The process described here instead utilizes an acid etch step, followed by serum incubation, to ensure that parylene-C regions become cell-adhesive, through a combination of reduction in hydrophobicity and serum protein binding.

The end result is a construct composed of two different substrates which, after biological activation, manifest respective cyto-adhesive or cyto-repulsive characteristics and so represents an effective cell pattering platform. Importantly, there is no need to introduce biological agents into the cleanroom facility as the patterned substrates can be stored indefinitely prior to use (whereupon they are activated using fetal bovine serum or another activation solution).

This parylene-C/SiO_2_ patterning platform is therefore a good candidate for a coalition with MEMS components, as the fabrication processes so closely mirror those used for microelectronic fabrication.

## Protocol

### 1. Fabrication of Parylene Patterns on SiO_2_: Process Flow (See Figure 1)

1. Design desired parylene-C configuration using a layout editor software package, capable of reading/writing CIF (Caltech Intermediate Form) or GDS-II (Graphic Database System-II) files. CIF and GDS-II are industry standard file formats for integrated circuit artwork layout.
2. Commission photo mask manufacture to an appropriate microelectronics facility, or make in-house if facilities exist.
3. Oxidize a silicon wafer in an atmospheric horizontal furnace (H_2_ 1.88 SLM and O_2_ 1.25 SLM) at 950 °C for 40 min to produce a 200 nm SiO_2_ layer (confirm thickness with a small spot spectroscopic reflectometer).
4. Prime the oxidized wafer with a silane adhesion promoter. Now deposit parylene-C at 22 °C at a rate of 1.298 nm/mg of dimer using a vacuum deposition system specifically designed to deposit parylene. A 100 nm thick parylene-C coating was used for all examples shown below.
5. Next deposit hexamethyldisilazane (HMDS) adhesion promoter on the parylene-coated wafer using a suitable photo-resist coating system.
6. Now spin the wafer at 4,000 rpm for 30 sec whilst applying positive photo-resist (resulting in a theoretical thickness of 1 μm), using the same photoresist coating system as above.
7. Soft bake the wafer for 60 sec at 90 °C.
8. Insert both the wafer and premanufactured photo mask into a mask aligner.
9. Expose the photoresist-coated wafer with a UV negative representation of the desired parylene-C configuration.
10. Bake exposed wafer for 60 sec at 110 °C.
11. Remove all exposed photo-resist from the wafer by developing in an appropriate developer solution.
12. Etch off the unprotected parylene. Use an oxygen plasma etch system (at a 50 mTorr chamber pressure, 49 sccm O_2_, 100 W RF power at 13.56 MHz, and an etch rate of 100 nm/min) to reveal the underlying SiO_2_.
13. Dice the wafer using an appropriate dicing saw (spindle speed 30,000 rpm, feed speed 7 mm/sec).
14. Rinse chips in deionized H_2_O and blow dry with nitrogen.
15. Store chips (indefinitely) in dust free boxes until required.

### 2. Chip Cleaning and Activation: Protocol

1. Remove residual photoresist from chips by washing in acetone for 10 sec.
2. Rinse in deionized distilled H_2_O 3x.
3. Make up fresh piranha acid (a 5:3 ratio of 30% hydrogen peroxide and 98% sulfuric acid). CAUTION: Prepare piranha acid with great care. It is an extremely powerful oxidizer, is strongly acidic, and mixing hydrogen peroxide with sulfuric acid is an exothermic reaction. Perform this stage in an acid fume hood. Allow 2 min to pass after mixing the piranha acid but use within 20 min.
4. Clean and etch chips by immersion in piranha acid for 10 min.
5. Rinse chips 3x in deionized H_2_O and transfer to a sterile culture dish.
6. Now activate chips for cell patterning. For example, add two chips per well to a 6-well plate and then add 2 ml of fetal bovine serum so as to fully immerse all chips. Perform this, and all subsequent cell culture stages, under sterile conditions in a laminar flow tissue culture hood.
7. Incubate chips in serum for 3-12 hr at 37 °C. NOTE: Steps 2.4-2.7 should be performed sequentially and without delay between steps. If serum activation is delayed by ≥24 hr after piranha-treatment, cell patterning is impaired due to reduced cell repulsion from SiO_2_ regions. Alternative activation solutions containing specific cell adhesion proteins can be used in place of fetal bovine serum. Such solutions alter the cell-adhesion characteristics of the two contrasting substrates (see representative results for an example).

### 3. Plating Cell Lines On-chip: Protocol

1. Remove chips from their activation solution and wash once for 10 sec in Hank's Balanced Salt Solution.
2. Place chip in a culture well and plate chosen cell type as a suspension in its usual growth media. Optimum cell plating density depends both on cell type and geometric pattern of parylene-C on-chip. A density of 5 x 10^4^ cells/ml is a sensible starting point.
3. Imaging is tailored to the underlying motivation for cell patterning but live cell behavior can easily be assessed using a dissecting microscope and a digital camera with a suitable relay lens.

## Representative Results

The photolithographic process of patterning SiO_2_ with parylene-C is illustrated in **Figure 1**. Once prepared, activation of chips in fetal bovine serum enables a wide range of cell types to be patterned in culture. Our group has successfully patterned primary murine hippocampal cells^14-16^, the HEK 293 cell line^17^, the human neuron-like teratocarcinoma (hNT) cell line^18^, primary murine cerebellar granule cells, and primary human glioma-derived stem-like cells.

**Figure 3** illustrates robust patterning of HEK 293 cells on a parylene pattern consisting of circular nodes with ‘cross-hair’ extensions. Chip activation in this example was with fetal bovine serum. By contrast, **Figure 4** illustrates the potential to augment the patterning platform by using alternative activation solutions. Using a solution of bovine serum albumin (3 mg/ml) and fibronectin (1 μg/ml) in HBSS, the previous patterning precept has been inverted.

**Figure 5** illustrates a different cell type (a primary human-derived stem-like cell line derived from a high grade glioma). Here, the geometry of the underlying pattern impacts cell behavior, with the pattern shown in **Figure 4A** promoting cell process growth along thin parylene-C tracks as shown in **Figures 4B **and** 4C**.

Some cell types do not pattern when using the established fetal bovine serum activation protocol. **Figure 6** illustrates 3T3 L1 cells growing to confluence with no discernable cyto-repulsive or cyto-adhesive difference between parylene-C and SiO_2_ regions.


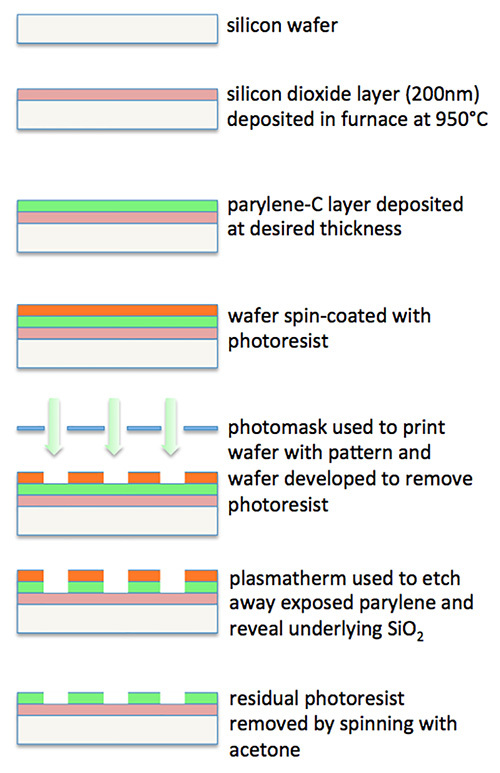
 **Figure 1. Flow diagram illustrating the process for fabrication of parylene-C patterns on SiO_2_.**


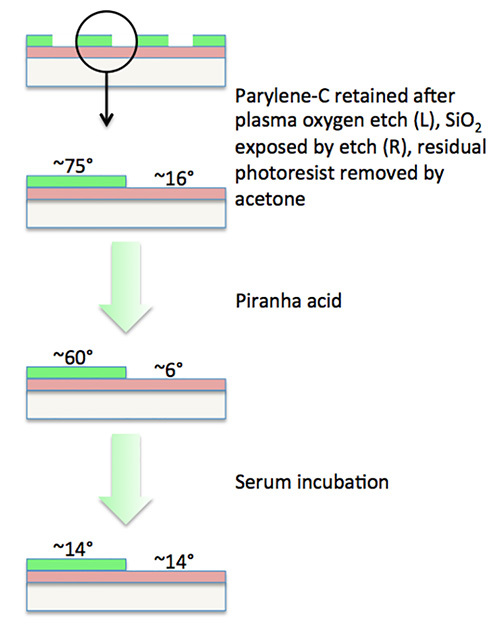
**Figure 2.** **Flow diagram illustrating the changes in contact angle for patterned parylene-C and SiO_2_ domains during chip activation steps.**


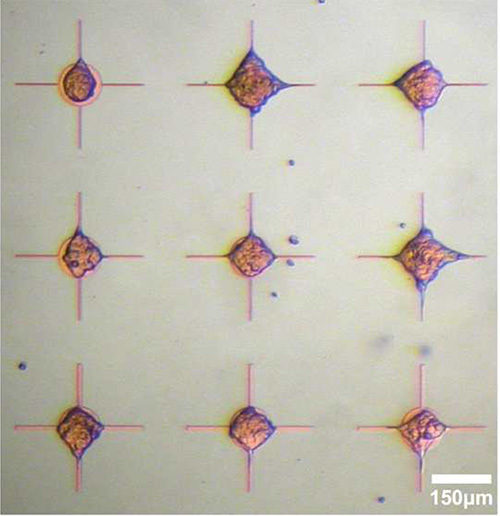
**Figure 3. Live cell imaging of HEK 293 cells cultured on parylene-C/SiO_2_ after three days *in vitro*. **Chips incubated for 3 hr in fetal bovine serum after which cells were plated in suspension at a concentration of 5 x 10^4^ cells/ml. Parylene-C promotes cell adhesion whilst bare SiO_2_ repels cells. Please click here to view a larger version of this figure.


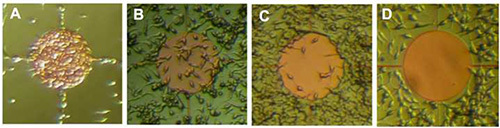
**Figure 4. Live cell imaging of HEK 293 cells cultured on parylene-C/SiO_2_ after three days *in vitro*.** Chips activated for 3 hr in different rationalized activation solutions: **A**: fetal bovine serum, **B**: vitronectin (1 μg/ml) in HBSS, **C**: Bovine serum albumin (3 mg/ml) + vitronectin (1 μg/ml) in HBSS, **D**: Bovine serum albumin (3 mg/ml) + fibronectin (1 μg/ml) in HBSS. Cells plated were in suspension at a density of 5 x 10^4^ cells/ml. Note how different treatment of the chip has resulted in reversal of the previous patterning dogma, with SiO_2_ now adhesive and parylene-C repulsive. Parylene-C node diameter 250 μm, adapted from Hughes *et al.*^17^ Please click here to view a larger version of this figure.


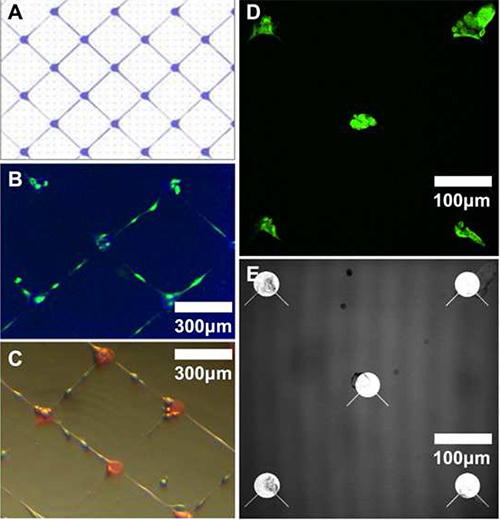
**Figure 5. Immunofluorescence images of primary human glioma-derived stem-like cells grown on various patterns of parylene-C on SiO_2_.A**: schematic illustrating the reticular parylene design shown in **B** and **C**. **B**: Fluorescence micrograph of fixed cells (after 4 days *in vitro*) stained for glial fibrillary acidic protein (GFAP). **C**: Light micrograph of live cells on same chip. **D**: Fluorescence image illustrating GFAP-stained cells on a different parylene design. **E**: Reflectance image of the node and spoke parylene design imaged in **D**. Please click here to view a larger version of this figure.


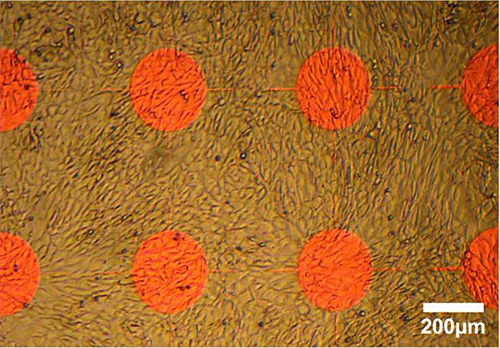
**Figure 6. Live cell imaging of 3T3 L1 cells cultured on parylene-C/SiO_2_ after four days *in vitro. ***Chips activated for 3 hr in fetal bovine serum after which cells were plated in suspension (3 x 10^4^ cells/ml). In this instance, the platform does not enable patterning, with cells becoming equally confluent on parylene-C and SiO_2_ regions. Please click here to view a larger version of this figure.

## Discussion

Immersion of chips in piranha acid serves not only to remove any residual organic material but also etches the substrate surfaces. This is key to enabling effective activation with fetal bovine serum. Failure to do so prevents cell-patterning and profoundly alters cell behavior on-chip. There is no requirement to sterilize chips after cleaning with piranha acid. Indeed sterilization by UV exposure has been shown to undermine cell patterning in a dose-dependent fashion^13^. Care must be taken to wash off all residual photoresist after the photolithographic process. Persisting photoresist can act as an unwanted cyto-adhesive layer that overrides patterning dictated by parylene-C/SiO_2_ geometry. Acetone is effective when using the photolithographic process described above and with the reagents specified. However, other types of photoresist may require a different solvent.

To assess the impact and success of the different fabrication steps, the contact angle of the two contrasting substrates can be measured. **Figure 2** illustrates the alterations that occur during the chip activation process. It is likely, however, that specific adhesive and repulsive protein components in serum ultimately enable the parylene-patterned chip to exert its respective cyto-adhesive or cyto-repulsive characteristics.

All representative results used chips with a parylene thickness of 100 nm, though we have successfully patterned using both thicker and thinner parylene layers. Importantly, this photolithographic etching technique allows much greater three-dimensional control of parylene configuration than that illustrated here. For example, using a combination of photomasks, it is possible to create parylene regions of mixed thickness. This opens the way to creating cell cultures with defined three-dimensional topography, going beyond simply dictating regions of cell adhesion/repulsion, potentially offering a means of integrating microfluidic channels into the construct.

As shown, however, this patterning platform is not universally effective across cell types. Different cell lines, with their varied cell adhesion molecule profiles, unsurprisingly behave differently when cultured on this platform. We have not yet identified the key components in serum, nor the complimentary cell-membrane receptors, which underpin this cell-patterning platform. Doing so in future promises to broaden its utility and specificity. For example, a ‘non-patterning’ cell line could be genetically modified to express the requisite adhesion molecule and so promote patterning.

## Disclosures

The authors declare no competing financial interests.
